# *In vitro* synchrotron-based radiography of micro-gap formation at the implant–abutment interface of two-piece dental implants

**DOI:** 10.1107/S0909049510001834

**Published:** 2010-02-03

**Authors:** A. Rack, T. Rack, M. Stiller, H. Riesemeier, S. Zabler, K. Nelson

**Affiliations:** a European Synchrotron Radiation Facility, Grenoble, France; b Charité, Department of Oral and Maxillofacial Surgery, Clinical Navigation and Robotics, Berlin, Germany; c Charité, Department of Maxillofacial and Facial-Plastic Surgery, Division of Oral Medicine, Radiology and Surgery, Berlin, Germany; d Bundesanstalt für Materialforschung und -prüfung, Division Structure Analysis, Polymer Analysis, Berlin, Germany; e Technical University of Berlin, Institute for Materials Engineering, Germany

**Keywords:** X-ray imaging, dental implants, digital radiography, implant–abutment interface

## Abstract

Micro-gap formation at the implant–abutment interface of two-piece dental implants was investigated *in vitro* using high-resolution radiography in combination with hard X-ray synchrotron radiation. Images were taken with the specimen under different mechanical loads of up to 100 N. The aim of this investigation was to prove the existence of micro-gaps for implants with conical connections as well as to study the mechanical behavior of the mating zone of conical implants during loading. Synchrotron-based radiography in comparison with classical laboratory radiography yields high spatial resolution in combination with high contrast even when exploiting micro-sized features in highly attenuating objects. The first illustration of a micro-gap which was previously indistinguishable by laboratory methods underlines that the complex micro-mechanical behavior of implants requires further *in vitro* investigations where synchrotron-based micro-imaging is one of the prerequisites.

## Abstract 2

Micro-radiography using hard X-ray synchrotron radiation is the first potential tool to allow an evaluation of the mechanical behavior of the dental implant–abutment complex during force application, thus enabling the enhancement of the design of dental implants which has been based on theoretical analysis to date.

## 1. Introduction

Two main designs of the dental implant have emerged within the last century, *i.e.* the two-piece and one-piece implant (Binon, 2000 ▶). To date, extensive research has been performed on the mechanisms of osseointegration of these implants and a high predictability of success for dental implants has been demonstrated (Albrektsson *et al.*, 2008 ▶). This status of knowledge obviously influenced a recent recognizable shift of research objectives to the composition of the implant components and their mating zone (Tsuge *et al.*, 2008 ▶; Semper *et al.*, 2009 ▶, 2010 ▶).

Two-piece implants consist of two separate components: the endosteal implant and the abutment carrying the prosthetic restoration connected by a screw joint (Binon, 2000 ▶). Unlike one-piece implants, two-piece implants are commonly used because they can be individually loaded with different types of abutments. Two-piece implants feature a mating zone in which the implant–abutment connection is ensured.

**Figure 1:**
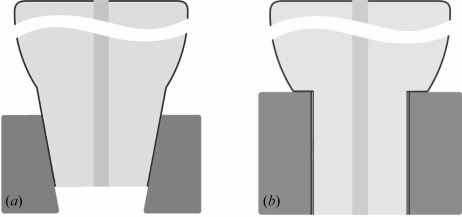
Two principles of mating zone utilized in all two-piece implants: based on (*a*) conical surfaces and (*b*) butt-joint connection.

The mating zone utilized in all two-piece implants can be differentiated into two principles: a butt-joint connection or one based on conical surfaces (*cf*. Fig. 1 ▶). The implant–abutment interface in butt-joint connections reveals a micro-gap (Jansen *et al.*, 1997 ▶; Coelho *et al.*, 2007 ▶). Adjacent to the micro-gap, when placed into the bone or gingiva, an inflammatory reaction has been described (Broggini *et al.*, 2006 ▶). The stimulus for this inflammatory reaction has been discussed to originate from the micro-gap (Broggini *et al.*, 2006 ▶; Hermann *et al.*, 2001 ▶). The micro-gap allows microbial colonization of the internal cavity of the implant–abutment complex as well as penetration of bacterial endotoxins into the surrounding tissue initiating a pathophysiological process that can result in bone loss and eventually implant loss (Jansen *et al.*, 1997 ▶; Broggini *et al.*, 2006 ▶; Steinebrunner *et al.*, 2005 ▶).

Direct observations of this micro-gap at the implant–abutment interface using X-rays are challenging owing to the limited resolution and contrast of the available laboratory-based methods: *in vivo* radiography and computed tomography are only applied to assess success and stability or failure of dental implants (see, for example, Yip *et al.*, 2004 ▶; Brägger, 1998 ▶). *In vitro* studies reported in the literature are very scarce and commonly limited to butt-joint connections where the micro-gap is visualized or its size estimated indirectly, *e.g.* *via* reference points (*cf.* Tsuge *et al.*, 2008 ▶; Coelho *et al.*, 2007 ▶). One approach to visualizing the micro-gap in both types of implant–abutment connection designs is by using micro-focus X-ray tubes for *in vitro* micro-radiography, where again only the gap in butt-joint connections was accessible (Zipprich *et al.*, 2007 ▶). Other methods where the micro-gap is commonly inspected after cyclic loading of butt-joint connections include scanning electron microscopy, optical microscopy, scanning laser microscopy or theoretical approaches *via* finite-element modeling (see, for example, Tsuge *et al.*, 2008 ▶; Coelho *et al.*, 2007 ▶; Hecker *et al.*, 2006 ▶). An *in vitro* observation of a micro-gap at the implant–abutment interface with conical-shaped connections has not been reported yet, hence even its non-existence was concluded due to this (Zipprich *et al.*, 2007 ▶). Recent leaking tests showed only the lack of sealing capability of this type of connection (Coelho *et al.*, 2008 ▶; Harder *et al.*, 2010 ▶).

In order to overcome limitations of the imaging technique we apply hard X-ray synchrotron radiation. The advances towards X-ray imaging using laboratory sources are the several orders of magnitude higher photon flux density available and the almost parallel beam propagation. This allows for extending the sample-to-source distance to up to more than 100 m and therefore to suppress the influence of the finite source size on the spatial resolution. The high monochromatic photon flux density increases the contrast while reducing artifacts. Synchrotron micro-imaging was established during the 1990s, and nowadays is available with spatial resolutions up to the sub-micrometer and time resolutions up to the microsecond range (Koch *et al.*, 1998 ▶; Rack *et al.*, 2009*a* ▶). Besides the improved resolution, imaging using synchrotron light sources also gives access to more sophisticated contrast modes like inline phase contrast or holo-tomography (Cloetens *et al.*, 1999 ▶); for further details see, for example, the book by Banhart (2008 ▶). Besides the numerous applications in materials science, archaeology or cultural heritage [*cf.* Baruchel *et al.* (2002 ▶, 2006 ▶) or Stock (2008 ▶)], the development is also approaching fast medical and even clinical applications (Keyrilainen *et al.*, 2008 ▶; Baruchel *et al.*, 2008 ▶; Stiller *et al.*, 2009 ▶; Issever *et al.*, 2008 ▶; Weitkamp *et al.*, 2008 ▶; Zabler *et al.*, 2006 ▶).

The purpose of this study is the *in vitro* visualization of a micro-gap formation at the implant–abutment interface with conical-shaped connection. The images taken show dimensions and the development of the micro-gap under different mechanical loads, knowledge which is important for understanding the functionality of implants with conical-shaped connection as well as to optimize and develop further their clinical applications.

## 2. Materials and methods

### 2.1. Dental implant and test stand

A virgin dental implant with a conical connection and a diameter of 4.1 mm (bone level implant, *L* = 14 mm, ref: 021.4114, lot: G6582; Straumann AG, Basel, Switzerland) and the corresponding rotation-safe abutment (NC-Mesosekundaerteil – Titan, ref: 022.2202, lot: F6601, Straumann AG, Basel, Switzerland) were assembled and screw-tightened with a torque of 0.25 Nm using the system-specific screw driver and ratchet. An individually fabricated steel-ball was glued to the abutment (Superglue X60; HBM Germany, Darmstadt) according to EN ISO Norm 14801:2003.

The implant–abutment assembly was embedded in an individually fabricated brass cylinder (Fraunhofer Institut Werkstoffmechanik, Freiburg, Germany) using Superglue X60 (HBM Germany, Darmstadt, Germany) according to EN ISO Norm 14801:2003; a crestal bone level 3 mm below the implant shoulder was simulated. The brass cylinder carrying the implant–abutment assembly was screw-fastened to an individually fabricated test stand made from stainless steel (V4A, Klaus Ellinger CNC Zerspannung GmbH, Berlin, Germany).

A static force (nominal 0 N, 30 N, 60 N, 100 N) was applied at a 90° angle to the implant axis onto the ball. The force application was monitored using a digital force gauge, model SH-500 [PCE-group OHG (serial No. 5808062790)].

### 2.2. Synchrotron-based micro-imaging

**Figure 2:**
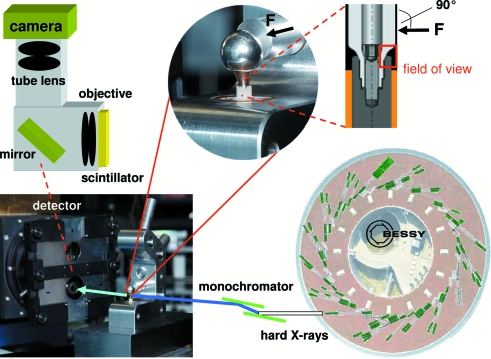
Sketch of the experimental set-up: the hard X-rays coming from an insertion device of the light source BESSY-II (right; Görner *et al.*, 2001 ▶; Rack *et al.*, 2008 ▶) are transmitted to the sample under load (photograph with zoom inset and sketch); the attenuated beam is converted into visible light by a scintillator screen. This luminescence image is captured *via* visible-light optics and a digital camera (Hartmann *et al.*, 1975 ▶). Only a small part of the specimen can be imaged owing to the detector’s limited field of view (*cf.* Fig. 3 ▶).

**Figure 3:**
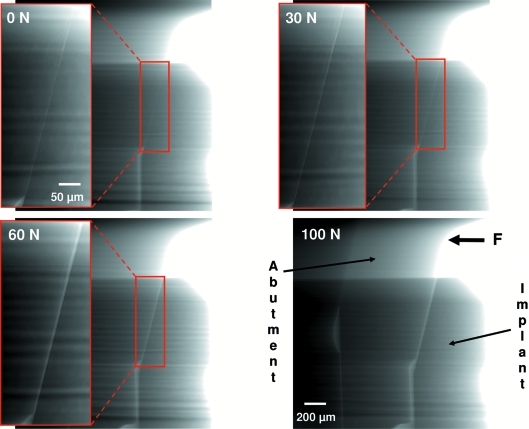
High-resolution radiographic images of the micro-gap formation at the implant–abutment interface for different mechanical load (the stripe modulations within the images originate from the X-ray monochromator used and are not a feature of the specimen). The relative position of this field of view with respect to the complete implant can be found in Fig. 2 ▶.

Measurements were carried out at the BAM*line* of the third-generation synchrotron light source BESSY-II (Helmholtz Zentrum Berlin für Materialien und Energie, Germany) (Görner *et al.*, 2001 ▶; Rack *et al.*, 2008 ▶). Numerous successful studies have already proven that this experimental station is excellently suited for synchrotron-based micro-imaging (see, for example, Kamenz & Weidemann, 2009 ▶; Rack *et al.*, 2009*b* ▶; Zabler *et al.*, 2007 ▶; Manke *et al.*, 2007 ▶). The white radiation from the wavelength-shifter insertion device of the BAM*line* was filtered with 0.2 mm Cu and 0.2 mm Be before passing through a double-multilayer monochromator which selected X-ray photons with an energy of 50 keV for imaging. The resulting photon flux density is of the order of 10^10^ photons s^−1^ mm^−2^ with an energy bandwidth of 1.7% (Rack *et al.*, 2008 ▶). Radiographic projection images were acquired using an indirect detector, based on the concept as introduced by Hartmann *et al.* (1975 ▶) as well as Bonse & Busch (1996 ▶); the luminescence image of a scintillator screen is optically coupled to a camera *via* diffraction-limited visible-light optics. A principle sketch of the detector design and the experimental set-up is displayed in Fig. 2 ▶. For this experiment, a 50 µm thin CdWO_4_ (CWO) single crystal glued on top of a 500 µm-thick undoped Y_3_Al_5_O_12_ (YAG) substrate was chosen as scintillator screen (Nagornaya *et al.*, 2005 ▶). The luminescence image of the crystal is read *via* a visible-light microscope, designed and manufactured by the company Optique Peter (Lyon, France): an Olympus objective Uplsapo (10×/0.4 NA) in combination with 2× eye-piece projects the image with an effective 20× magnification onto a CCD camera (0.43 µm effective pixel size; this value allows one to convert the size of the features in the images from pixels into meters). The diffraction-limited resolution of the objective is not reached owing to the thickness of the scintillator which exceeds the depth of focus of the objective. Hence, based on the thickness of the scintillator crystal we can estimate the spatial resolution of our detector system to be approximately 4 µm (250 line-pairs mm^−1^) (Koch *et al.*, 1998 ▶). The thickness of scintillator and substrate were required in order to protect the visible-light optics from radiation damage owing to the intense high-energy X-ray beam applied. As camera a pco.4000 (PCO AG, Germany) was used. The camera is based on a Kodak KAI-11000 interline transfer CCD chip with 4008 × 2672 pixels (each 9 µm in size); a dynamic range of 5000:1 was measured with exposure times between 0.1 s and 10 s, one signal unit (ADU) corresponds to a charge of 3 electrons in the corresponding potential well of the CCD chip, peak quantum efficiency above 50% at 500 nm. The field of view of the complete detector is approximately 1.7 mm × 1.1 mm. As the experiment is located 35 m away from the X-ray source (Rack *et al.*, 2008 ▶), the finite source size has no influence on the resolution (*e.g.* by penumbral blurring). The radiographic projection images shown in Fig. 3 ▶ were acquired with exposure times of 10 s. The distance between sample and detector was roughly 4 cm. Despite this relatively large propagation distance, common phase-contrast edge enhancements are only slightly present. This is due to the X-ray photon energy and spatial resolution of the employed indirect detector set-up, as well as the coherence properties of the BAM*line* (approximately 165 µm horizontal source size).

## 3. Results

The set of images taken during the *in vitro* measurement is shown in Fig. 3 ▶. The contrast is given by the X-ray absorption of the specimen which is determined roughly by its material and density as well as the effective thickness of the specimen along the X-ray beam path. The main components of the implant are marked and the position of the detector’s field of view with respect to the sample is sketched in Fig. 2 ▶. Furthermore, the orientation of the applied force *F* is also shown. Stripe modulations are present in all images which originate from the double-multilayer monochromator used; they are not a feature of the investigated sample. Owing to the limited field of view of a high-resolution indirect X-ray pixel detector, only a small part of the specimen is investigated. As the length of the gap is roughly 0.7 mm and the height of the synchrotron beam is limited, two images were acquired and later merged into one in order to illustrate the complete gap.

In all stages with different values for the applied force *F* (nominal 0 N, 30 N, 60 N, 100 N), a micro-gap between the abutment and the implant can be detected. The specimen as imaged without mechanical load (0 N) shows a micro-gap which is slightly below the resolution limit of our detector as it is only visible owing to a local reduction of the attenuation but not sampled by several pixels (*cf.* with the image at nominal 60 N mechanical load). Hence, we estimate its size to be in the range >1 µm and <<4 µm. For a nominal value of the applied force *F* = 30 N the micro-gap becomes clearly visible, sampled by between eight and ten pixels, so its size is in the range of 4 µm. When moving towards higher mechanical load, the gap at the implant–abutment interface opens further. At a nominal force *F* = 60 N, the size is around 11 µm (26 pixels) with the surfaces of the implant and the abutment running almost parallel. Finally, at a mechanical load of nominal *F* = 100 N, the micro-gap shows a non-parallel shape: at the upper end of the gap its size is roughly 22 µm (50 pixels) while at the lower end the size is around 15 µm (35 pixels).

## 4. Discussion

Animal studies have shown that the design of the implant–abutment connection has proven to be of high relevance for the stability of the soft and hard tissue surrounding the implant (Weng *et al.*, 2008 ▶). The exact mechanisms responsible for the biologic reaction of the bone in correlation to the micro-gap are still unclear. To date the illustration of the micro-gap in conical implant–abutment connections has not been feasible, as the conventional laboratory radiographic methods utilized did not allow distinct identification of clinically relevant gaps in the micrometer range (Zipprich *et al.*, 2007 ▶).

Implant–abutment assemblies are screwed joints that are exposed to dynamic loading owing to the masticatory process with axial and extra-axial forces (Binon, 2000 ▶). These forces can be up to 450 N, varying with the angle of application (Morneburg & Proeschel, 2002 ▶; Mericske-Stern *et al.*, 1992 ▶). Forces of up to 110 N applied at a 90° angle to the implant axis have been described to occur on the abutment carrying the implant-retained restorations (Mericske-Stern *et al.*, 1992 ▶). It has been shown with radiography using micro-focus X-ray tubes that butt-joint connections present an increase in micro-gap when extra-axial force is applied (Zipprich *et al.*, 2007 ▶).

Radiography using laboratory sources has been used to evaluate the micro-gaps of various systems but this method does not allow the detection of a micro-gap in internal conical implant–abutment connections. This is due to the limitations in resolution and contrast, given by the limited photon flux density, non-parallel beam propagation and influence of the finite source size in comparison with a synchrotron light source. The use of monochromatic hard X-ray synchrotron radiation to perform micro-radiography allowed for the first time the visualization of a micro-gap in internal conical implant–abutment joints.

The size of the micro-gap visualized varied, depending on the mechanical load, between approximately 1 µm and 22 µm, clearly ranging above the size for oral pathogens found responsible for a periimplantitis. The smallest size of oral bacteria found in the oral biofilm is 0.1 µm, whereas bacterial endotoxins with a size clearly smaller than 0.1 µm seem to be of importance. Endotoxins are lipopolysaccharide molecules (size of 10 kDa) found as part of the cell wall of gram-negative bacteria, and are released primarily upon cell lysis. These small-sized pathogenic molecules induce an inflammatory process within their vicinity (Broggini *et al.*, 2006 ▶). The size of the micro-gap has not been proven to correlate with the degree of inflammation; it is rather important that its existence seems to influence the periimplant environment (Hermann *et al.*, 2001 ▶).

As in all screwed joints the two mating components do not form a leak-proof tightness and have proven to show bacterial leakage in *in vitro* studies even without load application (Jansen *et al.*, 1997 ▶; Coelho *et al.*, 2007 ▶; Steinebrunner *et al.*, 2005 ▶; Harder *et al.*, 2010 ▶). Optical microscopy of implant–abutment connections based on a butt-joint principle have shown that there is a micro-gap of up to 10 µm with only punctual contact/fulcra of the mating zones which has been proposed to occur owing to the machining of the mating parts. To date there are no data available about the condition of the surface of the mating zone in conical dental implant joints (Coelho *et al.*, 2007 ▶). A continuous micro-gap without punctual contact of the mating components was seen within the unloaded specimen evaluated in this study. This location/site examined represents one cross section of the implant and shows that there is a missing surrounding surface contact. This incongruence allows for the correspondence between the external and internal environment of the screwed implant assembly even in unloaded conditions, as proposed in *in vitro* experiments (Jansen *et al.*, 1997 ▶). A possible explanation for this phenomenon is the imprecision of the machining of the parts which has been correlated to a repositioning instability of the abutment and to technical complications encountered in dental implant-retained restorations (Semper *et al.*, 2009 ▶, 2010 ▶; Jansen *et al.*, 1997 ▶; Coelho *et al.*, 2007 ▶). For generalization, this assumption needs further investigation with numerous samples, which has become accomplishable now by applying monochromatic hard X-ray synchrotron radiation.

Besides allowing for a precise illustration of the joint gap in conical dental implant–abutment connections by using monochromatic hard X-ray synchrotron radiation, a thorough investigation of the mechanical behavior in various loading situations of the components has become possible. Elucidation of the mode of the mechanical behavior of the implant–abutment joint under various loading scenarios regardless of their design will provide information to enhance the design and function of the joints and minimize the technical complications encountered to date.
